# The Linear Association Between A Body Shape Index and Abdominal Aortic Calcification in Individuals With Hypertension

**DOI:** 10.1002/clc.70349

**Published:** 2026-05-28

**Authors:** Xinyi Qiu, Zhenwei Wang

**Affiliations:** ^1^ Women's and Children's Clinical Medical College Fujian Medical University Fuzhou China; ^2^ Department of Cardiology The First Affiliated Hospital of Zhengzhou University Zhengzhou China

**Keywords:** A Body Shape Index, abdominal aortic calcification, hypertension, restricted cubic spline, subgroup analysis

## Abstract

**Objective:**

This study aimed to evaluate the association between A Body Shape Index (ABSI) and abdominal aortic calcification (AAC) in individuals with hypertension.

**Methods:**

This cross‐sectional analysis utilized data from 1486 participants in the National Health and Nutrition Examination Survey (NHANES). The relationship between ABSI and AAC was evaluated through logistic regression, subgroup analyses, receiver operating characteristic (ROC) curve assessment, and restricted cubic spline (RCS) modeling.

**Results:**

Multivariable logistic regression revealed that each standard deviation increase in ABSI corresponded to a 16.2% elevated AAC risk (OR = 1.162, 95% CI: 1.025–1.318, *p* = 0.019). Compared with the lowest quartile (Q1), individuals in Q3 and Q4 had significantly higher risks of AAC, with ORs of 1.554 (95% CI: 1.096–2.204, *p* = 0.013) and 1.503 (95% CI: 1.047–2.157, *p* = 0.027), respectively. Subgroup analyses revealed a robust association between ABSI and AAC that was consistently observed across strata. ROC curve analysis revealed that ABSI provided moderate discriminative ability for AAC detection in the overall cohort (AUC = 0.625, 95% CI: 0.596–0.654, *p* < 0.001), males (AUC = 0.632, 95% CI: 0.589–0.675, *p* < 0.001), and females (AUC = 0.627, 95% CI: 0.588–0.666, *p* < 0.001). RCS analysis, in both unadjusted and multivariable‐adjusted models, RCS analysis confirmed a dose‐dependent positive relationship between ABSI and AAC risk (P for nonlinearity > 0.05).

**Conclusion:**

Higher ABSI levels show a significant, independent association with increased AAC risk in hypertensive patients, suggesting potential clinical value as a indicator for early vascular calcification detection in this population.

## Graphical abstract

Higher A Body Shape Index (ABSI) was independently associated with increased abdominal aortic calcification (AAC) risk in hypertensive individuals and showed a linear positive relationship with AAC risk. ABSI may serve as a supplementary risk indicator, although its discriminative ability was limited.

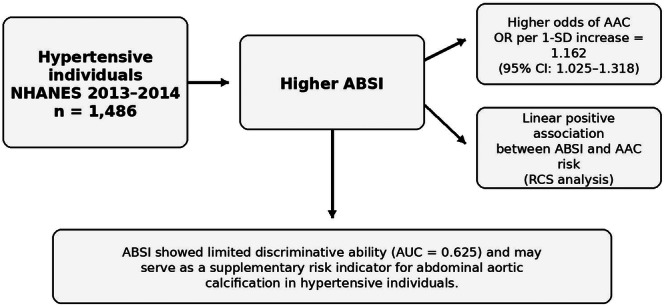

## 1 Introduction

Hypertension, affecting more than 1 billion individuals globally, persists as a major modifiable risk factor for cardiovascular disease and premature death worldwide [1]. World Health Organization (WHO) data indicate that an estimated 1.28 billion adults aged 30–79 years worldwide had hypertension in 2023, but about 46% remained unaware of their condition, and only 42% were receiving treatment, with just 21% achieving blood pressure control [2]. Hypertensive patients frequently develop cardiovascular complications such as left ventricular hypertrophy, myocardial infarction, heart failure, and abdominal aortic calcification (AAC). However, despite the wide array of available antihypertensive therapies, rates of uncontrolled hypertension continue to increase globally [3]. Epidemiological evidence consistently identifies hypertension as an independent predictor of AAC, with AAC presence conferring substantially elevated all‐cause mortality risk in this population [4, 5]. For instance, an NHANES‐based cohort study found that severe AAC (Kauppila score ≥ 6) was linked to a 70% higher all‐cause mortality risk among hypertensive individuals, with particularly strong associations observed in women and those with uncontrolled hypertension [4]. Therefore, identifying risk factors for AAC in hypertensive populations and implementing timely interventions are of critical importance.

A growing body of evidence has identified multiple predictors of AAC, including metabolic and inflammatory markers such as the atherogenic index of plasma (AIP), pan‐immune‐inflammation value (PIV), and neutrophil‐to‐lymphocyte ratio (NLR), all of which have been shown to be significantly associated with AAC or its severity in NHANES‐based studies [6, 7, 8]. Beyond traditional metabolic and inflammatory markers, body composition and fat distribution have emerged as key focus areas in cardiovascular disease research. Among the indices developed to quantify body shape, A Body Shape Index (ABSI) has emerged as a validated anthropometric index that adjusts waist circumference (WC) for height and weight, thereby reflecting central obesity and abdominal fat more precisely than BMI or WC alone [9]. Notably, in an analysis of NHANES 2013‐2014 data, Li et al. found that individuals with higher ABSI values (> 0.84) had a significantly increased prevalence of AAC, and ABSI remained independently associated with AAC after multivariable adjustment for potential confounders [10]. Relative to conventional anthropometric measures such as BMI and WC, ABSI demonstrated superior discriminatory power in predicting AAC, highlighting its clear advantage [10]. To date, the association between ABSI and AAC in hypertensive populations remains unexamined in peer‐reviewed literature.

Given this research gap, the present study aims to explore the association between ABSI and AAC specifically in hypertensive individuals. This study provides critical evidence to refine risk stratification paradigms and advance targeted preventive interventions for AAC in hypertensive patients.

## 2 Methods

### 2.1 Study Population

This cross‐sectional investigation analyzed data from the 2013–2014 cycle of the U.S. National Health and Nutrition Examination Survey (NHANES). The NHANES survey is a nationally representative, publicly available dataset that includes health and nutritional information from individuals across all demographics. Following the exclusion of participants lacking complete data on essential measurements including height, weight, waist circumference, hypertension status, and abdominal aortic calcification, the final analytical sample consisted of 1486 individuals. The National Center for Health Statistics Research Ethics Review Board granted ethical approval for this study. All participants provided written informed consent before enrollment. The research strictly followed the ethical guidelines established in the Declaration of Helsinki for human studies. To safeguard participant privacy, all collected data underwent complete anonymization prior to analysis.

### 2.2 Data Collection and Definitions

In this study, data were collected on demographics, anthropometric measurements, medical history, and laboratory indicators. Demographic variables included age, gender, race, and smoking status. Race was classified into five categories: Mexican American, Non‐Hispanic Black, Non‐Hispanic White, Other Hispanic, and Other races. Smoking was defined as current or former tobacco use, including individuals who smoked regularly or occasionally in the past and were still smoking at survey participation. Anthropometric and clinical data included height, weight, WC, BMI, diastolic blood pressure (DBP), and systolic blood pressure (SBP). Body mass index (BMI) was computed using the standard formula of weight in kilograms divided by height in meters squared (kg/m^2^). Hypertension was categorized as SBP ≥ 140 mmHg and/or DBP ≥ 90 mmHg, or current use of antihypertensive medication, based on established clinical criteria [11]. Medical history included the presence of diabetes, defined as a self‐reported diagnosis, fasting blood glucose (FBG) ≥ 7.0 mmol/L, glycated hemoglobin (HbA1c) ≥ 6.5%, or use of glucose‐lowering medications [12]. Laboratory indicators included a comprehensive panel: FBG, HbA1c, total cholesterol (TC), triglycerides, blood urea nitrogen (BUN), creatinine, high‐density lipoprotein cholesterol (HDL‐C), uric acid, total bilirubin, albumin, total calcium, vitamin D3, chloride, potassium, as well as complete blood count components such as hemoglobin, lymphocyte count, monocyte count, neutrophil count, white blood cell count (WBC), platelet count, mean corpuscular volume (MCV). All measurements were obtained following standardized NHANES protocols to ensure data accuracy and consistency.

### 2.3 Definition of ABSI

In this study, The ABSI was derived according to the following mathematical formula: ABSI = WC (cm)/[BMI (kg/m^2^)^(2/3)^× Height (cm)^(1/2)^] × 1000 [13]. Participants were categorized into four groups based on ABSI quartiles: Q1 ( ≤ 0.81, *n* = 371), Q2 (0.81–0.84, *n* = 372), Q3 (0.84–0.87, *n* = 372), and Q4 ( > 0.87, *n* = 371). Ascribable to the relatively small numerical values of ABSI, the values were multiplied by 100 for ease of analysis. Additionally, the standardized ABSI was calculated using the formula: Standardized ABSI = (Raw ABSI ‐ Mean)/Standard Deviation (SD), which was then applied to further statistical analyses to account for variations in ABSI distribution across the study population.

### 2.4 Assessment of Abdominal Aortic Calcification

AAC in this study was evaluated using dual‐energy X‐ray absorptiometry (DXA) [14]. Throughout the 2013–2014 NHANES data collection period, participants aged 40 years and older underwent lateral DXA scans of the thoracolumbar spine at Mobile Examination Centers (MECs). Individuals were excluded from scanning if they were pregnant, had received contrast agents such as barium within the previous 7 days, reported a body weight exceeding 450 pounds, or had a history of spinal instrumentation (e.g., Harrington rods).

Scans were performed using the Hologic Discovery Model A densitometer (Hologic Inc., Marlborough, MA) and analyzed with Apex version 3.2 software. AAC severity was evaluated through the AAC‐24 scoring system, a visual quantification method assessing calcification along both anterior and posterior aortic walls across vertebral segments L1‐L4. Each wall is divided into four segments, and calcification in each is scored as follows: 0 = no calcification, 1 = ≤ 1/3 of the wall, 2 = > 1/3 but ≤ 2/3, and 3 = > 2/3. Scores from the eight segments are summed, yielding a cumulative score range of 0 to 24.

To supplement this, the AAC‐8 scoring method was applied to assess the total length of visible calcification along the aorta from L1 to L4. Scores were assigned based on the extent of calcification relative to vertebral body height: 0 = none, 1 = ≤ 1 vertebral height, 2 = > 1 but ≤ 2, 3 = > 2 but ≤ 3, and 4 = > 3 vertebral heights.

For analytical purposes, Participants were stratified into two groups according to the presence or absence of AAC: those without AAC (score = 0, *n* = 910) and those with detectable AAC (*n* = 576).

### 2.5 Statistical Analysis

Statistical analyses were conducted using SPSS version 26.0 (IBM Corp) and R version 4.3.4 (R Foundation). The Shapiro‐Wilk test was employed to assess the normality of continuous variables, with non‐normally distributed variables reported as median and interquartile range (IQR). Group comparisons were performed using the Mann‐Whitney U test for two‐group analyses and the Kruskal‐Wallis test for comparisons across the four ABSI quartiles, while categorical variables were analyzed using the chi‐square test. Variables showing significant associations (*p* < 0.05) with AAC in univariate logistic regression were subsequently included in multivariable logistic regression models to control for potential confounders. Three models were used: Model 1 adjusted for age, Model 2 adjusted for age, race, and smoking, and Model 3 additionally adjusted for SBP, DBP, BUN, creatinine, total bilirubin, chloride, potassium, vitamin D3, lymphocyte count, monocyte count, MCV, MCHC, and platelet count. Subgroup analyses were conducted to explore the relationship between ABSI and AAC in various groups based on age (< 65 years or ≥ 65 years), BMI ( < 25 kg/m² or ≥ 25 kg/m²), diabetes status (yes or no), gender (male or female), HbA1c levels (< 6.5% or ≥ 6.5%), race (Mexican American, Other Hispanic, Non‐Hispanic White, Non‐Hispanic Black, or Other Races), SBP ( < 140 mmHg or ≥ 140 mmHg), and smoking status (yes or no). These subgroup analyses were considered exploratory in nature, and no formal adjustment for multiple comparisons was applied. The predictive performance of ABSI for AAC was evaluated through receiver operating characteristic (ROC) curve analysis, with area under the curve (AUC) calculations performed for both the overall population and gender‐stratified subgroups. In addition, comparative ROC analyses including BMI and WC were conducted using the same dataset to evaluate relative discriminative performance among different anthropometric indices. Restricted cubic splines (RCS) were employed to examine potential nonlinear relationships. Statistical significance was defined as a two‐sided *p*‐value < 0.05.

## 3 Results

### 3.1 Baseline Characteristics Stratified by Abdominal Aortic Calcification

Among the 1486 participants included in the analysis, 576 (38.8%) were diagnosed with AAC. As demonstrated in Table 1, Participants with AAC demonstrated significantly higher age and greater prevalence of Non‐Hispanic White ethnicity compared to those without AAC, while the proportions of Mexican Americans and non‐Hispanic Blacks were lower; they also had a higher prevalence of smoking, lower BMI, WC, and DBP, but higher SBP and ABSI scores (*p* < 0.05). Additionally, the AAC group showed elevated FBG, HbA1c, and decreased TC. Renal and hepatic function markers including bilirubin, BUN, creatinine, and uric acid were significantly higher, as were vitamin D3, potassium, and chloride levels (*p* < 0.05). Hematological differences included reduced lymphocyte count, elevated monocyte and neutrophil counts, increased MCV and MCH, and lower platelet count (*p* < 0.05), whereas no statistically significant differences were found in gender distribution, diabetes status, triglycerides, HDL‐C, albumin, or calcium. Comparisons in Table 1 were descriptive and were not adjusted for multiple testing.

**TABLE 1:** Baseline characteristics of grouping according to AAC.

Variables	Total population	Non‐AAC	AAC	P value
N	1486	910	576	
Age, years	63.00 (54.00, 72.00)	60.00 (51.00, 67.00)	69.00 (61.00, 77.00)	< 0.001
Gender, n (%)				0.984
Male	679 (45.7)	416 (45.7)	263 (45.7)	
Female	807 (54.3)	494 (54.3)	313 (54.3)	
Race, n (%)				< 0.001
Mexican American	164 (11.0)	114 (12.5)	50 (8.7)	
Other Hispanic	127 (8.5)	85 (9.3)	42 (7.3)	
Non‐Hispanic White	662 (44.5)	343 (37.7)	319 (55.4)	
Non‐Hispanic Black	368 (24.8)	261 (28.7)	107 (18.6)	
Other Races	165 (11.1)	107 (11.8)	58 (10.1)	
Smoking, n (%)	729 (49.1)	413 (45.4)	316 (54.9)	< 0.001
Diabetes, n (%)	445 (29.9)	268 (29.5)	177 (30.7)	0.600
BMI, kg/m^2^	28.70 (25.80, 33.10)	29.80 (26.40, 34.40)	27.90 (24.83, 31.30)	< 0.001
SBP, mmHg	131.33 (120.00, 143.33)	129.33 (118.67, 141.33)	133.33 (122.00, 146.67)	< 0.001
DBP, mmHg	71.33 (64.00, 78.67)	73.33 (66.25, 80.67)	69.33 (60.67, 75.33)	< 0.001
Waist circumference, cm	101.90 (93.60, 111.10)	103.35 (94.20,112.83)	99.80 (92.40, 107.80)	< 0.001
ABSI*100	83.77 (80.61, 87.02)	82.98 (79.84, 86.00)	85.02 (81.95, 87.94)	< 0.001
Fasting blood glucose, mg/dL	101.00 (91.00, 117.00)	100.00 (90.00, 113.00)	103.00 (92.00, 122.75)	0.001
HbA1c, %	5.80 (5.40, 6.23)	5.80 (5.40, 6.20)	5.90 (5.50, 6.30)	0.026
Total cholesterol, mg/dL	192.00 (163.00, 217.00)	194.00 (165.00, 218.00)	188.00 (159.00, 214.00)	0.024
Triglycerides, mg/dL	139.50 (93.00, 203.25)	141.00 (89.00, 204.25)	139.00 (99.00, 201.00)	0.617
HDL‐C, mg/dL	51.00 (42.00, 61.00)	51.00 (42.00, 61.00)	51.00 (42.00, 61.00)	0.625
Blood urea nitrogen, mg/dL	14.00 (11.00, 18.00)	14.00 (11.00, 17.00)	15.00 (12.00, 20.00)	< 0.001
Creatinine, mg/dL	0.92 (0.77, 1.09)	0.90 (0.76, 1.05)	0.94 (0.78, 1.14)	< 0.001
Uric acid, mg/dL	5.60 (4.80, 6.70)	5.50(4.70, 6.60)	5.60 (4.90, 6.80)	0.044
Total bilirubin, mg/dL	0.60 (0.50, 0.70)	0.60 (0.50, 0.70)	0.60 (0.50, 0.80)	0.036
Albumin, g/dL	4.20 (4.00, 4.40)	4.20 (4.00, 4.40)	4.20 (4.00, 4.40)	0.761
Total calcium, mg/dL	9.50 (9.30, 9.70)	9.50 (9.28, 9.70)	9.50 (9.30, 9.70)	0.199
Vitamin D3, nmol/L	64.80 (45.50, 84.60)	63.10 (42.88, 79.90)	66.30 (48.53, 91.45)	< 0.001
Chlorine, mmol/L	104.00 (102.00, 106.00)	104.00 (102.00, 106.00)	104.00 (102.00, 106.00)	< 0.001
Potassium, mmol/L	4.00 (3.80, 4.30)	4.00 (3.80, 4.20)	4.00 (3.80, 4.30)	0.002
WBC, 10^9^/L	7.10 (5.80, 8.40)	7.10 (5.70, 8.40)	7.10 (5.90, 8.60)	0.190
Lymphocyte count, 10^9^/L	2.00 (1.60, 2.50)	2.09 (1.60, 2.60)	2.00 (1.50, 2.50)	0.013
Monocyte count, 10^9^/L	0.60 (0.50, 0.70)	0.60 (0.50, 0.70)	0.60 (0.50, 0.70)	< 0.001
Neutrophil count, 10^9^/L	4.10 (3.30, 5.10)	4.10 (3.20, 5.00)	4.22 (3.30, 5.30)	0.024
Hemoglobin, g/dL	13.80 (12.80, 14.70)	13.90 (12.90, 14.80)	13.80 (12.70, 14.68)	0.172
Mean corpuscular volume, fL	89.90 (86.50, 93.20)	89.60 (86.10, 92.40)	90.90 (87.43, 94.30)	< 0.001
MCH, pg	30.40 (29.10, 31.70)	30.30 (28.90, 31.40)	30.80 (29.30, 32.08)	< 0.001
Platelet count, 10^9^/L	226.00 (192.00, 265.00)	231.00 (198.00, 271.00)	219.00 (182.00, 256.00)	< 0.001

Abbreviations: AAC, abdominal aortic calcification; BMI, body mass index; SBP, systolic blood pressure; DBP, diastolic blood pressure; ABSI, a body shape index; HbA1c, glycosylated hemoglobin; HDL‐C, high‐density lipoprotein cholesterol; WBC, white blood cell count; MCH, mean corpuscular hemoglobin.

### 3.2 Baseline Characteristics According to ABSI Quartiles

As shown in Supporting Information S1: Table 1, the prevalence of AAC increased markedly with rising ABSI quartiles, from 24.0% in Q1% to 36.3% in Q2, 43.0% in Q3, and reaching 51.8% in Q4 (*p* < 0.001). Participants in higher ABSI quartiles were significantly older, more likely to be male, non‐Hispanic White, and smokers, and had a higher prevalence of diabetes (all *p* < 0.001). With increasing ABSI, BMI and DBP decreased, whereas WC increased (*p* < 0.001). Metabolic indicators such as FBG, HbA1c, and triglycerides were significantly elevated across ABSI quartiles, while HDL‐C declined (*p* < 0.001). Renal function markers including BUN, creatinine, and uric acid also increased significantly, as did vitamin D3, and potassium levels (*p* < 0.05). In hematologic indices, WBC, monocyte, and neutrophil counts, along with MCV and MCH, were significantly higher in the upper ABSI groups, while platelet counts were lower (*p* < 0.001). Variables such as SBP, hemoglobin, albumin, calcium, and total bilirubin did not differ significantly between quartiles.

### 3.3 Multivariable Logistic Regression Analysis of ABSI and Abdominal Aortic Calcification

As shown in Table 2, both continuous and standardized ABSI demonstrated a significant positive association with elevated risk of AAC across all models. When analyzed as a continuous measure, each 1‐unit increase in ABSI×100 showed a statistically significant relationship with a higher odds of AAC in Model 1 (OR = 1.043, 95% CI: 1.018–1.068, *p* = 0.001), and the association remained statistically significant after adjustment for demographic and clinical covariates in Model 2 (OR = 1.031, 95% CI: 1.006–1.057, *p* = 0.016) and Model 3 (OR = 1.031, 95% CI: 1.005–1.057, *p* = 0.019). Similarly, for each 1‐SD increase in standardized ABSI, the odds of AAC increased significantly in Model 1 (OR = 1.232, 95% CI: 1.091–1.392, *p* = 0.001), and the association persisted robust in Model 2 (OR = 1.165, 95% CI: 1.029–1.319, *p* = 0.016) and Model 3 (OR = 1.162, 95% CI: 1.025–1.318, *p* = 0.019). When analyzed categorically, individuals in the highest ABSI quartile (Q4) demonstrated significantly elevated odds of AAC relative to those in the lowest quartile (Q1), even after full adjustment in Model 3 (OR = 1.503, 95% CI: 1.047–2.157, *p* = 0.027); Correspondingly, the third quartile (Q3) also showed statistically significant relationships with AAC (OR = 1.554, 95% CI: 1.096–2.204, *p* = 0.013).

**TABLE 2:** Multivariate logistic regression analysis of ABSI and AAC.

Variables	Model 1			Model 2			Model 3		
	OR	95% CI	P value	OR	95% CI	P value	OR	95% CI	P value
As a continuous variable									
ABSI*100	1.043	1.018–1.068	0.001	1.031	1.006–1.057	0.016	1.031	1.005–1.057	0.019
Standardized ABSI	1.232	1.091–1.392	0.001	1.165	1.029–1.319	0.016	1.162	1.025–1.318	0.019
As a classification variable									
Q1	Ref								
Q2	1.376	0.983–1.928	0.063	1.334	0.948–1.879	0.098	1.391	0.981–1.973	0.064
Q3	1.699	1.216–2.372	0.002	1.533	1.091–2.153	0.014	1.554	1.096–2.204	0.013
Q4	1.750	1.243–2.463	0.001	1.498	1.055–2.128	0.024	1.503	1.047–2.157	0.027
P for trend			0.005			0.070			0.072

*Note*: Model 1: adjusted for age; Model 2: adjusted for age, race, and smoking; Model 3: adjusted for age, race, smoking, systolic blood pressure, diastolic blood pressure, blood urea nitrogen, creatinine, total bilirubin, chlorine, potassium, vitamin D3, lymphocyte count, monocyte count, mean corpuscular volume, mean corpuscular hemoglobin, and platelet count.

Abbreviations: ABSI, a body shape index; AAC, abdominal aortic calcification; OR, odds ratio; CI, confidence interval.

Notably, given the large number of laboratory and clinical covariates included in Model 3, a multicollinearity assessment was conducted using the variance inflation factor (VIF). Results indicated that all covariates had VIF values well below commonly accepted thresholds (all < 5) (Supporting Information S1: Table 2).

### 3.4 Subgroup Association Between ABSI and Abdominal Aortic Calcification

In the subgroup analysis (Table 3), after adjusting for all confounding variables in the multivariable logistic regression analysis, the risk of AAC in females was 1.887, 1.641, and 1.742 times higher in Q2, Q3, and Q4 versus Q1. Additionally, each 0.01‐unit increase and each SD increase in ABSI was associated with a 3.9% and 21.3% increase in AAC risk, respectively. Among participants aged < 65 years, the AAC risk in Q2 and Q3 was 1.578 and 1.602 times higher than in Q1. In those aged ≥ 65 years, Q3 and Q4 showed 2.065‐ and 2.472‐fold increased risks compared to Q1, with each 0.01‐unit and SD increase in ABSI associated with a 6.1% and 34.6% higher risk of AAC, respectively. In non‐Hispanic Whites, Q3 was associated with a 1.786‐fold increased risk of AAC compared to Q1. Among individuals of other races, Q2 and Q4 had 3.971‐ and 5.134‐fold higher AAC risks than Q1, respectively. In the smoking subgroup, Q3 showed a 2.018‐fold higher AAC risk compared to Q1. Among participants without diabetes, Q3 and Q4 had 2.034‐ and 1.885‐fold increased risks, with each 0.01‐unit and SD increase in ABSI linked to 4.9% and 26.8% greater AAC risk, respectively. In individuals with BMI < 25.0 kg/m^2^, AAC risk in Q3 and Q4 was 4.991 and 3.696 times higher than in Q1, and each 0.01‐unit and SD increase in ABSI was associated with a 9.2% and 55.5% rise in AAC risk, respectively. In those with SBP ≥ 140 mmHg, Q2, Q3, and Q4 showed 1.979‐, 1.987‐, and 2.703‐fold higher AAC risks, with each 0.01‐unit and SD increase in ABSI associated with 6.5% and 37.1% increased risk, respectively. Among participants with HbA1c < 6.5%, Q3 and Q4 had 1.731‐ and 1.938‐fold higher AAC risks, and every 0.01‐unit and SD increase in ABSI was linked to a 5.0% and 27.6% higher AAC risk, respectively.

**TABLE 3:** Subgroup association between ABSI and AAC.

	Q2 versus Q1		Q3 versus Q1		Q4 versus Q1		ABSI*100		Standardized ABSI	
	OR (95% CI)	P value	OR (95% CI)	P value	OR (95% CI)	P value	OR (95% CI)	P value	OR (95% CI)	P value
Gender										
Male	0.862 (0.482‐1.545)	0.619	1.633 (0.943‐2.828)	0.080	1.507 (0.852‐2.667)	0.159	1.030 (0.987‐1.076)	0.174	1.162 (0.936‐1.442)	0.174
Female	1.887 (1.218‐2.925)	0.004	1.641 (1.041‐2.588)	0.033	1.742 (1.094‐2.775)	0.019	1.039 (1.006‐1.074)	0.019	1.213 (1.032‐1.426)	0.019
Age										
< 65 years	1.578 (1.005‐2.478)	0.048	1.602 (1.011‐2.537)	0.045	1.451 (0.857‐2.456)	0.166	1.027 (0.990‐1.066)	0.150	1.145 (0.952‐1.377)	0.150
≥ 65 years	1.664 (0.970‐2.855)	0.064	2.065 (1.214‐3.511)	0.007	2.472 (1.481‐4.126)	0.001	1.061 (1.025‐1.098)	0.001	1.346 (1.134‐1.599)	0.001
Race										
Mexican American	1.682 (0.492‐5.749)	0.407	0.970 (0.289‐3.251)	0.960	0.603 (0.143‐2.542)	0.491	0.962 (0.873‐1.061)	0.438	0.825 (0.507‐1.341)	0.438
Other Hispanic	1.709 (0.465‐6.277)	0.419	2.840 (0.645‐12.496)	0.167	1.755 (0.407‐7.566)	0.451	1.078 (0.966‐1.204)	0.181	1.457 (0.840‐2.527)	0.181
Non‐Hispanic White	1.170 (0.668‐2.051)	0.583	1.786 (1.063‐3.002)	0.029	1.357 (0.794‐2.320)	0.264	1.028 (0.993‐1.054)	0.123	1.146 (0.964‐1.363)	0.123
Non‐Hispanic Black	1.150 (0.585‐2.260)	0.685	1.202(0.572‐2.526)	0.627	1.883 (0.883‐4.015)	0.102	1.037 (0.979‐1.098)	0.211	1.201 (0.901‐1.599)	0.211
Other Race	3.971 (1.136‐13.882)	0.031	2.441 (0.614‐9.700)	0.205	5.134 (1.348‐19.550)	0.016	1.097(0.986‐1.220)	0.089	1.586 (0.932‐2.701)	0.089
Smoking										
Yes	1.524 (0.928‐2.503)	0.096	2.018(1.244‐3.274)	0.004	1.525 (0.932‐2.496)	0.093	1.031 (0.994‐1.059)	0.100	1.165 (0.971‐1.396)	0.100
No	1.258 (0.767‐2.063)	0.363	1.211 (0.730‐2.008)	0.459	1.620 (0.953‐2.754)	0.074	1.043 (1.018‐1.068)	0.001	1.029 (0.992‐1.067)	0.125
Diabetes										
Yes	1.158 (0.550‐2.441)	0.699	0.996 (0.462‐2.020)	0.927	0.892 (0.423‐1.882)	0.764	0.997 (0.951‐1.045)	0.905	0.986 (0.779‐1.247)	0.905
No	1.464 (0.986‐2.172)	0.059	2.034 (1.367‐3.026)	0.001	1.885 (1.214‐2.862)	0.003	1.049 (1.018‐1.081)	0.002	1.268 (1.091‐1.475)	0.002
BMI										
< 25.0 kg/m^2^	1.953 (0.830‐4.596)	0.125	4.991 (2.088‐11.929)	0.001	3.696 (1.583‐8.630)	0.003	1.092 (1.030‐1.158)	0.003	1.555 (1.162‐2.081)	0.003
≥ 25 kg/m^2^	1.312 (0.892‐1.931)	0.168	1.195 (0.811‐1.761)	0.367	1.147 (0.761‐1.728)	0.513	1.009 (0.980‐1.038)	0.562	1.044 (0.903‐1.205)	0.562
SBP										
< 140 mmHg	1.179 (0.779‐1.786)	0.436	1.465 (0.968‐2.217)	0.071	1.247 (0.809‐1.923)	0.318	1.071 (0.986‐1.049)	0.293	1.087 (0.931‐1.269)	0.293
≥ 140 mmHg	1.979 (1.036‐3.780)	0.039	1.987 (1.052‐3.753)	0.034	2.703 (1.424‐5.130)	0.002	1.065 (1.019‐1.113)	0.005	1.371 (1.100‐1.708)	0.005
HbA1c										
< 6.5%	1.400 (0.956‐2.050)	0.084	1.731 (1.186‐2.527)	0.004	1.938 (1.309‐2.870)	0.001	1.050 (1.020‐1.080)	0.001	1.276 (1.107‐1.471)	0.001
≥ 6.5%	1.022 (0.410‐2.547)	0.962	1.061 (0.412‐2.730)	0.903	0.448 (0.170‐1.183)	0.105	0.954 (0.900‐1.011)	0.111	0.789 (0.589‐1.056)	0.111

*Note*: Subgroup analysis adjusted for age, race, smoking, systolic blood pressure, diastolic blood pressure, blood urea nitrogen, creatinine, total bilirubin, chlorine, potassium, vitamin D3, lymphocyte count, monocyte count, mean corpuscular volume, mean corpuscular hemoglobin, and platelet count.

Abbreviations: ABSI, a body shape index; AAC, abdominal aortic calcification; BMI, body mass index; SBP, systolic blood pressure; HbA1c, glycosylated hemoglobin; OR, odds ratio; CI, confidence interval.

### 3.5 Predictive Value of ABSI for AAC

**FIGURE 1:**
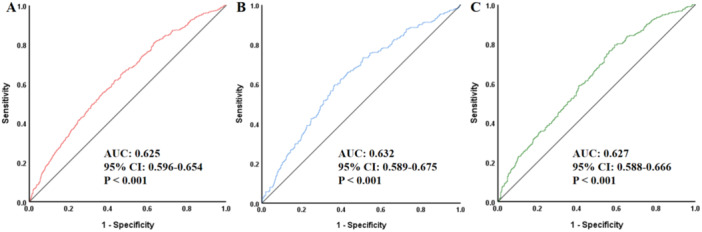
ROC curves of ABSI for predicting AAC in the total population (A), males (B), and females (C). ROC, receiver operating characteristic; AUC, area under the curve; CI, confidence interval; ABSI, a body shape index; AAC, abdominal aortic calcification.

As shown in ROC analysis of Figure 1, ABSI demonstrated a certain predictive ability for AAC in the total population (A), males (B), and females (C). Specifically, the AUC was 0.625 (95% CI: 0.596–0.654, *p* < 0.001) in the total population, 0.632 (95% CI: 0.589–0.675, *p* < 0.001) in males, and 0.627 (95% CI: 0.588–0.666, *p* < 0.001) in females. However, all AUC values were below 0.7, indicating limited discriminative performance. Although statistically significant, this level of predictive ability was generally considered insufficient for clinical prediction when used alone. To further contextualize these findings, we additionally compared ABSI with other commonly used anthropometric indices (BMI and WC), and the results were presented in Supporting Information S1: Figure 1. ABSI showed an AUC of 0.625 (95% CI: 0.596–0.654, *p* < 0.001), which was slightly higher than that of BMI (AUC: 0.613, 95% CI: 0.585–0.642, *p* < 0.001) and WC (AUC: 0.572, 95% CI: 0.543–0.602, *p* < 0.001). However, all indices demonstrated only modest predictive ability overall.

### 3.6 Linear Association Between ABSI and AAC

**FIGURE 2:**
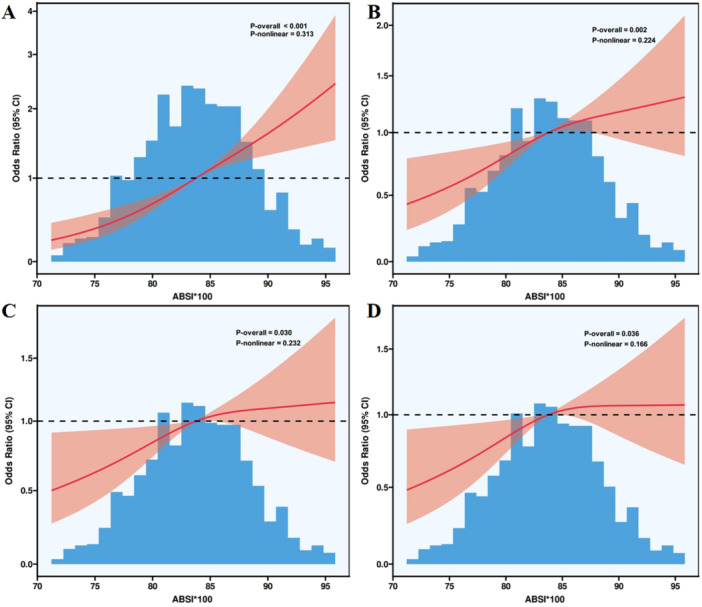
RCS plots illustrating the association between ABSI and the risk of AAC. A: unadjusted model; B: Model 1 (adjusted for age); C: Model 2 (adjusted for age, race, and smoking status); D: Model 3 (further adjusted for systolic blood pressure, diastolic blood pressure, blood urea nitrogen, creatinine, total bilirubin, chlorine, potassium, vitamin D3, lymphocyte count, monocyte count, mean corpuscular volume, mean corpuscular hemoglobin, and platelet count). RCS, restricted cubic spline; CI, confidence interval; ABSI, a body shape index; AAC, abdominal aortic calcification.

As shown in Figure 2, RCS analyses across all models—including the unadjusted model (A), partially adjusted models (B and C), and the fully adjusted model (D)—demonstrated a significant positive association between ABSI and the risk of AAC. In each model, the P‐overall values were all ≤ 0.05, while the P‐nonlinear values remained > 0.05, indicating that the relationship between ABSI and AAC risk followed a linear dose–response pattern rather than a nonlinear one.

## 4 Discussion

This represents the first cross‐sectional investigation systematically examining the ABSI‐AAC relationship in hypertensive populations. Our analyses demonstrated a significant, independent positive association between increasing ABSI levels and elevated AAC risk that persisted after comprehensive multivariable adjustment. The robustness of this association was substantiated through consistent findings across all prespecified subgroups. Restricted cubic spline analysis confirmed a linear dose‐response relationship. However, given the modest effect size, these findings should be interpreted as indicative of an association rather than a clinically actionable relationship. Moreover, ABSI demonstrated statistically significant but limited predictive ability for AAC. Taken together, these results suggest that ABSI may serve as a supplementary risk indicator rather than a clinically definitive tool for AAC risk stratification in hypertensive patients.

Previous studies have confirmed the association between various nutrition‐related indicators, such as WC, waist‐to‐hip ratio (WHR), visceral fat area, and weight‐adjusted waist index (WWI), and the risk of AAC [15, 16, 17]. As an illustration, in a cross‐sectional study conducted by Ozturk et al. at Kecioren Training and Research Hospital in Ankara, Turkey, 316 patients undergoing CT scans for urolithiasis were evaluated [18]. Among them, 127 individuals (40.2%) were found to have AAC, and patients with AAC had significantly greater visceral fat area, total abdominal fat, WC, and waist‐to‐hip ratio, while their psoas muscle area was notably lower compared to those without AAC. Notably, individuals with a visceral fat area exceeding 131 cm^2^ had a 4.5‐fold increased risk of AAC. Additionally, AAC severity was significantly correlated with liver and spleen density, suggesting that visceral adiposity, waist measurements, and muscle mass may serve as useful predictors of AAC. Besides, in a study utilizing NHANES 2013–2014 data, Qin et al. analyzed 3082 adults aged 40 years and older to investigate the association between the WWI and AAC [19]. WWI demonstrated a significant positive association with AAC scores (β = 0.34), and its association with SAAC showed a nonlinear pattern. Notably, at WWI levels below 11.11, each unit increment was significantly associated with elevated SAAC risk (OR = 2.86), while no significant association was observed beyond this threshold. These results suggest that WWI demonstrates potential as a practical and reliable indicator for AAC risk stratification in individuals aged 40 years and older. Furthermore, in a study of 931 men with a mean age of 63.7 years, Higo et al. explored the correlation between various anthropometric and CT‐derived obesity indicators and aortic artery calcification (AoAC) [15]. They found that 82.6% of participants had evidence of AoAC, and multivariate analysis revealed that higher ratios of visceral to subcutaneous fat (VAT/SAT) and visceral to total fat (VAT/TAT) were significantly associated with the presence of AoAC, indicating that the distribution of abdominal fat—particularly the relative amount of visceral fat—measured by CT is more strongly related to AoAC than general measures of obesity. Notably, a recent large‐scale cross‐sectional study using 2013–2014 NHANES data by Yin et al. included 1,062 participants to examine the associations between novel anthropometric indices—ABSI and body roundness index (BRI)—and the prevalence of AAC and SAAC [16]. The findings revealed a U‐shaped relationship between ABSI and the risk of AAC and SAAC, indicating that the risk increased with ABSI up to a certain point before declining. In contrast, BRI showed a consistent positive association with both AAC and SAAC risk. Furthermore, higher ABSI and BRI values were linked to greater AAC severity, and ROC analysis demonstrated that ABSI had stronger predictive accuracy for AAC than BRI, suggesting that ABSI may serve as a more sensitive indicator for identifying individuals at higher risk of AAC. Additionally, the findings by Li et al. regarding the association between ABSI and AAC further support the results of the present study [10]. Utilizing NHANES data, their analysis similarly revealed a significant positive association between elevated ABSI levels and AAC risk, with consistent effects observed across all demographic subgroups. Interestingly, participants with AAC often exhibited higher ABSI values despite having relatively lower WC. This phenomenon can be explained by the fact that ABSI adjusts WC for both height and weight, allowing it to more accurately capture abnormal abdominal fat distribution and visceral adiposity than WC alone. Such a pattern may reflect the presence of a “metabolically obese normal weight” phenotype, in which individuals have a normal or lower overall body weight but a disproportionately high visceral fat burden. In hypertensive populations, this phenotype may be particularly relevant, as excess visceral fat can exacerbate vascular inflammation, endothelial dysfunction, and metabolic disturbances, all of which contribute to the development and progression of AAC. In addition, ABSI showed superior discriminative ability for predicting AAC relative to traditional anthropometric measures such as BMI and WC, indicating that it may serve as a more sensitive indicator of fat distribution abnormalities that are closely linked to vascular calcification. Importantly, these prior studies were predominantly conducted in the general population, and the magnitude of association observed in our study appears broadly comparable to those previously reported, suggesting that the effect size of ABSI on AAC risk may not be substantially modified by hypertension status alone. Therefore, the added clinical value of focusing on hypertensive populations should be interpreted cautiously, and may primarily lie in contextualizing existing evidence rather than establishing a distinct effect.

Compared with previous studies conducted mainly in the general population, this investigation offers multiple notable methodological advantages. First, it is the first to focus specifically on individuals with hypertension, a population inherently at higher risk for vascular calcification. Given that hypertension is independently associated with both vascular remodeling and calcification processes, examining ABSI within this context may provide additional insights into risk stratification in a clinically vulnerable population, although the observed associations do not appear fundamentally different in magnitude from those reported in the general population. Second, the association between ABSI and AAC was confirmed to be robust in hypertensive patients through comprehensive multivariable adjustment and extensive subgroup analyses. However, it should be noted that the inclusion of a relatively large number of covariates in the fully adjusted model, primarily selected based on univariate statistical significance, may introduce potential collinearity and over‐adjustment bias, particularly if some variables are interrelated or lie along the causal pathway between ABSI and AAC. This may, to some extent, attenuate or obscure the true association. Nevertheless, we acknowledge that extensive adjustment and subgroup analyses, while improving robustness, may also complicate the interpretability of the core findings. Besides, given the exploratory nature of these subgroup analyses and the absence of adjustment for multiple comparisons, these findings should be interpreted with caution. In addition, we observed that the proportion of males increased across ABSI quartiles, whereas no significant difference in sex distribution was found between AAC groups. This apparent discrepancy may be explained by the fact that ABSI incorporates WC normalized for height and weight, and males generally have greater height and absolute WC, leading to higher ABSI values independent of adiposity alone. Furthermore, ABSI is considered to better reflect visceral fat distribution, and males are more prone to visceral fat accumulation than females, providing a biologically plausible explanation for the observed association between ABSI and sex. However, AAC is a multifactorial condition influenced by age, metabolic status, inflammation, and blood pressure, which may attenuate the direct impact of sex‐specific fat distribution patterns on AAC prevalence. Therefore, although ABSI is associated with sex, this does not necessarily translate into corresponding sex differences in AAC risk. These findings suggest that sex may act as a potential confounding or modifying factor, and thus the observed associations should be interpreted with caution. Third, we employed a RCS model to demonstrate a clear linear positive relationship between ABSI and AAC risk. This linear pattern differs from the U‐shaped association reported in some general population studies, which may suggest potential population‐specific differences, although this requires cautious interpretation. Fourth, we quantitatively evaluated the predictive value of ABSI for AAC using ROC analysis. Although ABSI showed statistically significant predictive ability, the AUC values were modest (all < 0.7), indicating limited discriminative performance. Furthermore, comparison with BMI and WC demonstrated only marginal differences, suggesting that ABSI does not substantially outperform traditional anthropometric measures. These findings indicate that ABSI is unlikely to be useful as a standalone clinical screening or diagnostic tool, and its role may be limited to a complementary indicator within broader risk assessment frameworks. Besides, the observed linear association in this hypertensive population suggests that the pathophysiological mechanisms linking ABSI to AAC risk may differ from those in the general population, warranting further investigation. At present, hypertension‐specific biological mechanisms underlying this association remain insufficiently elucidated, and our findings should be interpreted primarily as epidemiological evidence rather than mechanistic confirmation. Moreover, given the cross‐sectional design of this study, causal inference cannot be established, and it remains unclear whether ABSI‐based risk stratification would lead to improvements in clinical management or patient outcomes.

Although this study established an independent association between ABSI and the risk of AAC in individuals with hypertension, the underlying biological mechanisms remain to be fully elucidated. The following interpretations are mainly based on existing literature and are not directly supported by the present study, and thus should be interpreted with caution. First, ABSI is considered a more accurate indicator of visceral fat accumulation and central obesity than BMI [17]. Excess VAT has been linked to metabolic dysregulation and chronic low‐grade inflammation through the release of pro‐inflammatory mediators [20], which have been associated with endothelial dysfunction and vascular injury in previous studies [21, 22, 23, 24]. In our study, individuals with AAC exhibited lower BMI and WC but higher ABSI values, which may suggest that ABSI captures aspects of body composition not reflected by conventional measures; however, this remains speculative. Supporting evidence from animal studies shows that transplantation of visceral fat can accelerate atherosclerosis [22]. Second, elevated ABSI is often associated with other risk factors for vascular calcification, such as diabetes [23], CKD, and dyslipidemia. For example, ABSI is positively correlated with LDL‐C levels, and elevated LDL‐C has been implicated in vascular calcification [25, 26, 27]. These conditions may contribute through mechanisms such as hyperglycemia‐induced advanced glycation end‐product formation, disturbed mineral metabolism, and oxidative stress [28, 29, 30]. Taken together, ABSI may reflect a combination of visceral adiposity–related metabolic and inflammatory states; however, these interpretations remain hypothetical and should be considered hypothesis‐generating rather than mechanistic conclusions.

The present study has several limitations. First, although it included a relatively large sample size, all participants were derived from a specific time frame of a U.S. national epidemiological survey. Therefore, the generalizability of the findings to other ethnic populations may be limited, and future large‐scale, multicenter studies are needed to validate the association between ABSI and the risk of AAC in hypertensive populations across diverse populations. Second, the diagnosis of hypertension in this study was primarily based on self‐reported questionnaire data, which may be subject to recall bias. Future analyses using clinical data from healthcare institutions are warranted to confirm these findings. Third, ABSI was assessed only once at baseline without follow‐up or dynamic monitoring, making it impossible to evaluate the long‐term exposure or temporal changes in ABSI and their impact on AAC development. Prospective clinical studies are required to explore the association between longitudinal or time‐varying ABSI levels and AAC risk in hypertensive individuals. Fourth, as a retrospective observational study, it lacked genetic data, limiting the ability to conduct Mendelian randomization or other genetic analyses to infer causality. Future studies incorporating genetic information are necessary to establish potential causal links between ABSI and AAC. Fifth, although this study adjusted for a wide range of baseline variables, other potentially important factors such as genetic susceptibility, environmental exposures, dietary patterns, and occupational health status were not included, which may weaken the strength of the evidence. Moreover, despite adjustment for multiple covariates, residual confounding cannot be completely excluded, particularly due to unmeasured or unavailable variables. In addition, the covariate selection strategy based on univariate analysis may lack a prespecified conceptual framework, and some included variables may represent intermediate factors rather than true confounders, thereby potentially introducing over‐adjustment bias. Future studies should consider more parsimonious and clinically guided modeling strategies to improve interpretability and causal inference. Furthermore, the subgroup analyses were exploratory and were not adjusted for multiple comparisons, which may increase the risk of type I error. Sixth, given that age increases significantly across ABSI quartiles, the potential correlation between ABSI and age may introduce residual confounding despite multivariable adjustment, and thus the observed associations should be interpreted with caution. Seventh, the baseline comparisons in Table 1 included multiple laboratory and clinical variables without adjustment for multiple testing, which may increase the likelihood of spurious statistically significant findings; this highlights that Table 1 analyses are primarily descriptive and should be interpreted with caution rather than as evidence for causal relationships. Therefore, these findings should be interpreted with caution and regarded as hypothesis‐generating rather than confirmatory. Eighth, although the validated AAC‐24 scoring system was used to assess AAC, the outcome in the primary analysis was dichotomized into the presence versus absence of AAC. This approach may lead to information loss and reduced statistical power, and it may limit the ability to fully capture variations in AAC severity. However, the main analysis of this study retained a binary outcome (presence/absence of AAC) based on the following considerations: first, this classification method has been widely used in previous NHANES‐based studies and provides clear clinical interpretability, allowing for straightforward distinction of the presence of vascular calcification, which represents a clinically meaningful state; second, a dichotomous outcome is more suitable for evaluating risk associations using logistic regression models, and the results are more intuitive and easier to interpret in a clinical context. Additionally, the core objective of this study was to examine the association between ABSI and the risk of AAC (i.e., the presence of AAC), rather than the continuous variation in AAC severity. Furthermore, the AAC scores in this study population were somewhat skewed, and modeling them as a continuous variable would require additional data transformation or assumption checks for linear regression. Future studies could consider modeling the AAC‐24 score as a continuous variable, for example using linear regression or other methods appropriate for skewed distributions, to provide a more comprehensive assessment of the relationship between ABSI and AAC severity, thereby capturing risk gradients and potential dose‐response patterns more accurately. Despite these limitations, the findings provide meaningful insights and lay the groundwork for further investigation.

## 5 Conclusion

In conclusion, higher levels of ABSI demonstrated significant positive associations with elevated AAC risk in hypertensive patients, with consistent effects observed across all subgroup analyses. Furthermore, a potential dose‐dependent positive association between ABSI and AAC risk was confirmed. However, given the modest discriminative performance and the cross‐sectional nature of the study, ABSI should be considered as a supplementary indicator rather than an independent clinical screening tool. These findings are primarily associative and hypothesis‐generating, and further large‐scale, multicenter prospective studies should validate these findings and explore ABSI‐AAC associations across more diverse demographics.

## Author Contributions

**Xinyi Qiu:** conceptualization, methodology, software, investigation, data curation, formal analysis, visualization, writing – original draft, writing – review and editing. **Zhenwei Wang:** conceptualization, writing – review and editing, validation, funding acquisition, project administration, supervision. All authors read and approved the final manuscript.

## Ethics Statement

This study was based on publicly available data from the National Health and Nutrition Examination Survey (NHANES), which is conducted by the National Center for Health Statistics (NCHS). All NHANES protocols were approved by the NCHS Research Ethics Review Board, and written informed consent was obtained from all participants. The present analysis complied with the ethical standards of the Declaration of Helsinki.

## Consent

The authors have nothing to report.

## Conflicts of Interest

The authors declare no conflicts of interest.

## Supporting information


**Supporting File:** clc70349‐sup‐0001‐Supplementary_materials.docx.

## Data Availability

The Journal of Clinical Hypertension (JCH). The datasets analyzed in this study are publicly available from the NHANES website (https://wwwn.cdc.gov/nchs/nhanes/default.aspx).
