# Supplementary material for: The Linear Association Between A Body Shape Index and Abdominal Aortic Calcification in Individuals With Hypertension
Source: Clin Cardiol. 2026 May 28;49(6):e70349. doi: 10.1002/clc.70349 (PMC13239843; doi:10.1002/clc.70349)
Supplement: Supplementary file 1 — Supporting File: clc70349‐sup‐0001‐Supplementary_materials.docx. [file CLC-49-e70349-s001.docx]

| **Table S1.** Baseline characteristics according to ABSI quartile grouping | | | | | |
| --- | --- | --- | --- | --- | --- |
| Variables | Q1 | Q2 | Q3 | Q4 | P value |
| N | 371 | 372 | 372 | 371 |  |
| Age, years | 55.00 (48.00, 65.00) | 63.00 (52.25, 70.00) | 63.00 (55.00, 72.00) | 69.00 (63.00, 78.00) | < 0.001 |
| Gender, n (%) |  |  |  |  | < 0.001 |
| Male | 114 (30.7) | 162 (43.5) | 198 (53.2) | 205 (55.3) |  |
| Female | 257 (69.3) | 210 (56.5) | 174 (46.8) | 166 (44.8) |  |
| Race, n (%) |  |  |  |  | < 0.001 |
| Mexican American | 42 (11.3) | 40 (10.8) | 48 (12.9) | 34 (8.2) |  |
| Other Hispanic | 34 (9.2) | 41 (11.0) | 24 (6.5) | 28 (7.5) |  |
| Non-Hispanic White | 135 (36.4) | 137 (36.9) | 185 (49.7) | 205 (55.3) |  |
| Non-Hispanic Black | 122 (32.9) | 103 (27.7) | 76 (20.4) | 67 (18.1) |  |
| Other Races | 38 (10.2) | 51 (13.7) | 39 (10.5) | 37 (10.0) |  |
| Smoking, n (%) | 155 (41.8) | 173 (46.5) | 189 (50.8) | 212 (57.1) | < 0.001 |
| Diabetes, n (%) | 72 (19.4) | 107 (28.8) | 128 (34.4) | 138 (37.2) | < 0.001 |
| BMI, kg/m^2^ | 30.60 (26.60, 35.30) | 28.65 (25.73, 33.10) | 28.70 (26.40, 33.10) | 27.80 (24.80, 31.00) | < 0.001 |
| SBP, mmHg | 129.33 (119.33, 140.67) | 131.33 (120.67, 144.00) | 131.33 (118.67, 144.00) | 132.67 (122.00, 148.00) | 0.052 |
| DBP, mmHg | 73.33 (66.00, 81.33) | 72.00 (66.00, 78.67) | 62.00 (54.00, 71.33) | 61.33 (53.6, 69.33) | < 0.001 |
| Waist circumference, cm | 97.40 (89.40, 106.60) | 99.29 (90.83, 108.95) | 104.00 (97.50, 114.70) | 105.90 (97.20, 115.60) | < 0.001 |
| ABSI*100 | 78.20 (76.46, 79.60) | 82.30 (81.34, 83.04) | 85.31 (84.48, 86.01) | 88.99 (87.78, 90.95) | < 0.001 |
| Fasting blood glucose, mg/dL | 97.00 (90.00, 108.00) | 101.00 (90.00, 115.75) | 104.00 (91.00, 119.00) | 104.00 (93.00, 125.00) | < 0.001 |
| HbA1c, % | 5.70 (5.30, 6.00) | 5.80 (5.40, 6.20) | 5.80 (5.50, 6.40) | 5.90 (5.50, 6.50) | < 0.001 |
| Total cholesterol, mg/dL | 195.00 (169.00, 219.00) | 190.50 (165.00, 215.00) | 191.00 (160.00, 220.75) | 188.00 (156.00,2 12.00) | 0.058 |
| Triglycerides, mg/dL | 126.00 (84.00, 176.00) | 136.00 (89.25, 198.25) | 153.50 (101.25, 220.50) | 151.00 (99.00, 212.00) | < 0.001 |
| HDL-C, mg/dL | 54.00 (45.00, 64.00) | 53.50 (43.25, 63.00) | 48.00 (40.00, 58.00) | 49.00 (41.00, 59.00) | < 0.001 |
| Blood urea nitrogen, mg/dL | 13.00 (11.00, 16.00) | 14.00 (11.00, 18.00) | 14.00 (11.25, 18.00) | 15.00 (12.00, 20.00) | < 0.001 |
| Creatinine, mg/dL | 0.86 (0.73, 1.04) | 0.90 (0.77, 1.05) | 0.94 (0.79, 1.11) | 0.94 (0.80, 1.14) | < 0.001 |
| Uric acid, mg/dL | 5.50 (4.60, 6.40) | 5.60 (4.70, 6.60) | 5.70 (4.83, 6.70) | 5.70 (4.90, 6.90) | 0.002 |
| Total bilirubin, mg/dL | 0.60 (0.40, 0.70) | 0.60 (0.50, 0.70) | 0.60 (0.50, 0.78) | 0.60 (0.50, 0.80) | 0.233 |
| Albumin, g/dL | 4.20 (4.00, 4.40) | 4.20 (4.00, 4.40) | 4.20 (4.00, 4.40) | 4.20 (3.90, 4.40) | 0.101 |
| Total calcium, mg/dL | 9.50 (9.30, 9.70) | 9.50 (9.20, 9.70) | 9.50 (9.30, 9.70) | 9.50 (9.20, 9.70) | 0.834 |
| Vitamin D3, nmol/L | 64.20 (42.70, 85.40) | 62.55 (44.45, 80.38) | 63.35 (43.15, 83.20) | 65.50 (49.70, 90.50) | 0.021 |
| Chlorine, mmol/L | 104.00 (102.00, 106.00) | 104.00 (102.00, 106.00) | 104.00 (102.00, 106.00) | 104.00 (102.00, 106.00) | 0.834 |
| Potassium, mmol/L | 4.00 (3.70, 4.20) | 4.00 (3.80, 4.30) | 4.10 (3.80, 4.30) | 4.10 (3.80, 4.40) | < 0.001 |
| WBC, 10^9^/L | 6.90 (5.50, 8.10) | 6.80 (5.70, 8.30) | 7.14 (5.90, 8.60) | 7.20 (6.30, 8.80) | < 0.001 |
| Lymphocyte count, 10^9^/L | 2.09 (1.60, 2.60) | 2.00 (1.60, 2.40) | 2.00 (1.60, 2.50) | 2.00 (1.50, 2.40) | 0.124 |
| Monocyte count, 10^9^/L | 0.58 (0.40, 0.60) | 0.54 (0.40, 0.70) | 0.60 (0.50, 0.70) | 0.60 (0.50, 0.80) | < 0.001 |
| Neutrophil count, 10^9^/L | 3.80 (3.00, 4.70) | 4.00 (3.10, 4.88) | 4.22 (3.40, 5.10) | 4.40 (3.50, 5.80) | < 0.001 |
| Hemoglobin, g/dL | 13.70 (12.80, 14.40) | 13.70 (12.80, 14.60) | 13.90 (12.90, 14.90) | 13.90 (12.70, 14.90) | 0.056 |
| Mean corpuscular volume, fL | 89.70 (86.20, 92.60) | 89.78 (86.33, 92.90) | 89.85 (86.63, 93.18) | 91.20 (87.10, 93.90) | 0.001 |
| MCH, pg | 30.30 (29.00, 31.30) | 30.30 (28.80, 31.50) | 30.38 (29.20, 31.68) | 30.80 (29.30, 31.68) | 0.001 |
| Platelet count, 10^9^/L | 231.00 (203.00, 275.00) | 230.00 (195.00, 266.50) | 225.50 (189.75, 263.75) | 216.00 (182.00, 258.00) | < 0.001 |
| AAC, n (%) | 89 (24.0) | 135 (36.3) | 160 (43.0) | 192 (51.8) | < 0.001 |

ABSI, a body shape index; BMI, body mass index; SBP, systolic blood pressure; DBP, diastolic blood pressure; HbA1c, glycosylated hemoglobin; HDL-C, high-density lipoprotein cholesterol; WBC, white blood cell count; MCH, mean corpuscular hemoglobin; AAC, abdominal aortic calcification.

**Table S2.** Assessment of multicollinearity

| Variables | VIF | Tolerance |
| --- | --- | --- |
| Standardized ABSI | 1.104 | 0.821 |
| Age | 1.351 | 0.548 |
| Race | 1.046 | 0.914 |
| Smoking | 1.042 | 0.921 |
| Systolic blood pressure | 1.169 | 0.732 |
| Diastolic blood pressure | 1.224 | 0.667 |
| Blood urea nitrogen | 1.354 | 0.546 |
| Creatinine | 1.272 | 0.618 |
| Total bilirubin | 1.059 | 0.892 |
| Chlorine | 1.046 | 0.914 |
| Potassium | 1.072 | 0.871 |
| Vitamin D3 | 1.091 | 0.84 |
| Lymphocyte count | 1.14 | 0.769 |
| Monocyte count | 1.102 | 0.823 |
| Mean corpuscular volume | 2.126 | 0.221 |
| Mean corpuscular hemoglobin | 2.129 | 0.22 |
| Platelet count | 1.095 | 0.833 |

ABSI, a body shape index; VIF, variance inflation factors.


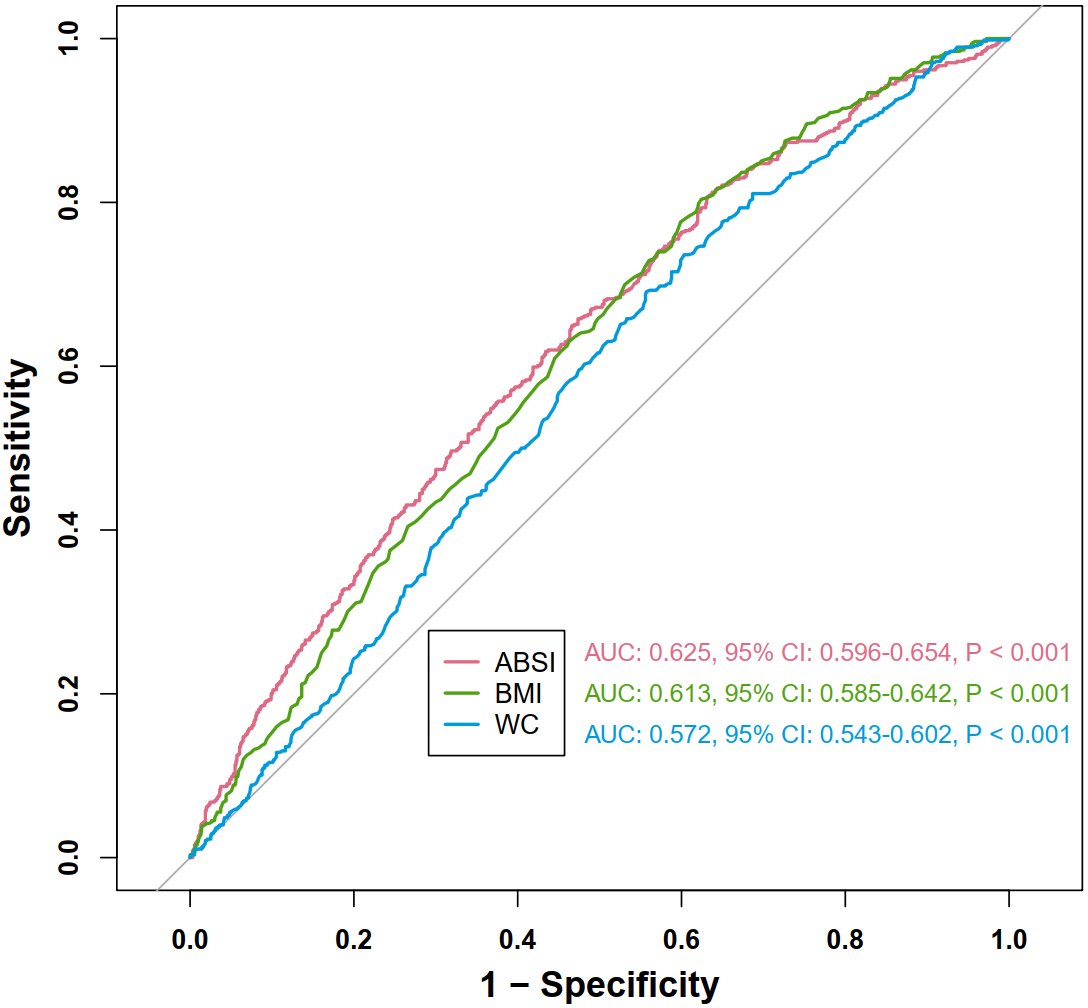


**Figure S1.** Comparative ROC curves of ABSI, BMI, and WC for predicting AAC.

ROC, receiver operating characteristic; BMI, body mass index; WC, waist circumference; AUC, area under the curve; CI, confidence interval; ABSI, a body shape index; AAC, abdominal aortic calcification.
